# Mental health response to COVID-19 in China and impact on psychiatrists

**DOI:** 10.1192/j.eurpsy.2021.106

**Published:** 2021-08-13

**Authors:** M. Zhao

**Affiliations:** Shanghai Mental Health Center, Shanghai Jiao Tong University School of Medicine, Shanghai, China

**Keywords:** Mental health needs, online service, expert consensus, triage strategy

## Abstract

The COVID-19 outbreak has raised numerous challenges for mental health service system in China. The pandemic has many affects on clinical, research and teaching, due to the strict quarantine in china. Fight the COVID-19 became the most important thing in work. We outlined major mental health needs during COVID-19 outbreak from the exiting studies and challenges for mental health professionals, and how to manage these challenges in China. To reduce the risk of negative psychological outcomes associated with the COVID-19 pandemic, the Central Health Authority of China and different national academic societies have integrated mental health crisis interventions into the general deployment of disease prevention and treatment. The NHCC developed a mental health triage strategy to provide four levels of psychological crisis interventions. More than 20 specific guidelines and expert consensus for mental health services for the COVID-19 outbreak were disseminated by the end of February 2020 to provide timely guidance for frontline health care professionals. External mental health expert teams in other provinces were also established to provide emergency mental health services in Hubei province, China. In addition, widespread adoption of online public education, psychological counseling, and hotline services have been set up for those in need. Although the COVID-19 pandemic has been under control in China, we should take a proactive lead to share its protocol of emergency mental health services with other countries affected by the COVID-19 pandemic. Also international cooperation is urgely needed to control the COVID-19 pandemic worldwide. Large-scale epidemiological surveys should be conducted to examine the prevalence of mental health problems associated with the COVID-19 pandemic to inform the development of appropriate mental health services in future.

**Disclosure:**

No significant relationships.

